# Dynamic changes of DNA methylation induced by benzo(a)pyrene in cancer

**DOI:** 10.1186/s41021-023-00278-1

**Published:** 2023-07-01

**Authors:** Huizeng Wang, Bingchun Liu, Hong Chen, Peixin Xu, Huiting Xue, Jianlong Yuan

**Affiliations:** 1 Department of Laboratory Medicine, the Affiliated Hospital of Inner Mongolia Medical University, Hohhot, 010050 China; 2 Stem Cell Research Center, the Affiliated Hospital of Inner Mongolia Medical University, Hohhot, 010050 China; 3 College of Basic Medicine, Inner Mongolia Medical University, Hohhot, 010010 China

**Keywords:** Benzo(a)pyrene, Cancer, DNA methylation, Tumor suppressor gene, Proto-oncogene

## Abstract

Benzo(a)pyrene (BaP), the earliest and most significant carcinogen among polycyclic aromatic hydrocarbons (PAHs), has been found in foods, tobacco smoke, and automobiles exhaust, etc. Exposure to BaP induced DNA damage directly, or oxidative stress-related damage, resulting in cell apoptosis and carcinogenesis in human respiratory system, digestive system, reproductive system, etc. Moreover, BaP triggered genome-wide epigenetic alterations by methylation, which might cause disturbances in regulation of gene expression, and thereby induced cancer. It has been proved that BaP reduced genome-wide DNA methylation, and activated proto-oncogene by hypomethylation in the promoter region, but silenced tumor suppressor genes by promoter hypermethylation, resulting in cancer initiation and progression. Here we summarized the changes in DNA methylation in BaP exposure, and revealed the methylation of DNA plays a role in cancer development.

**Graphical Abstract:**

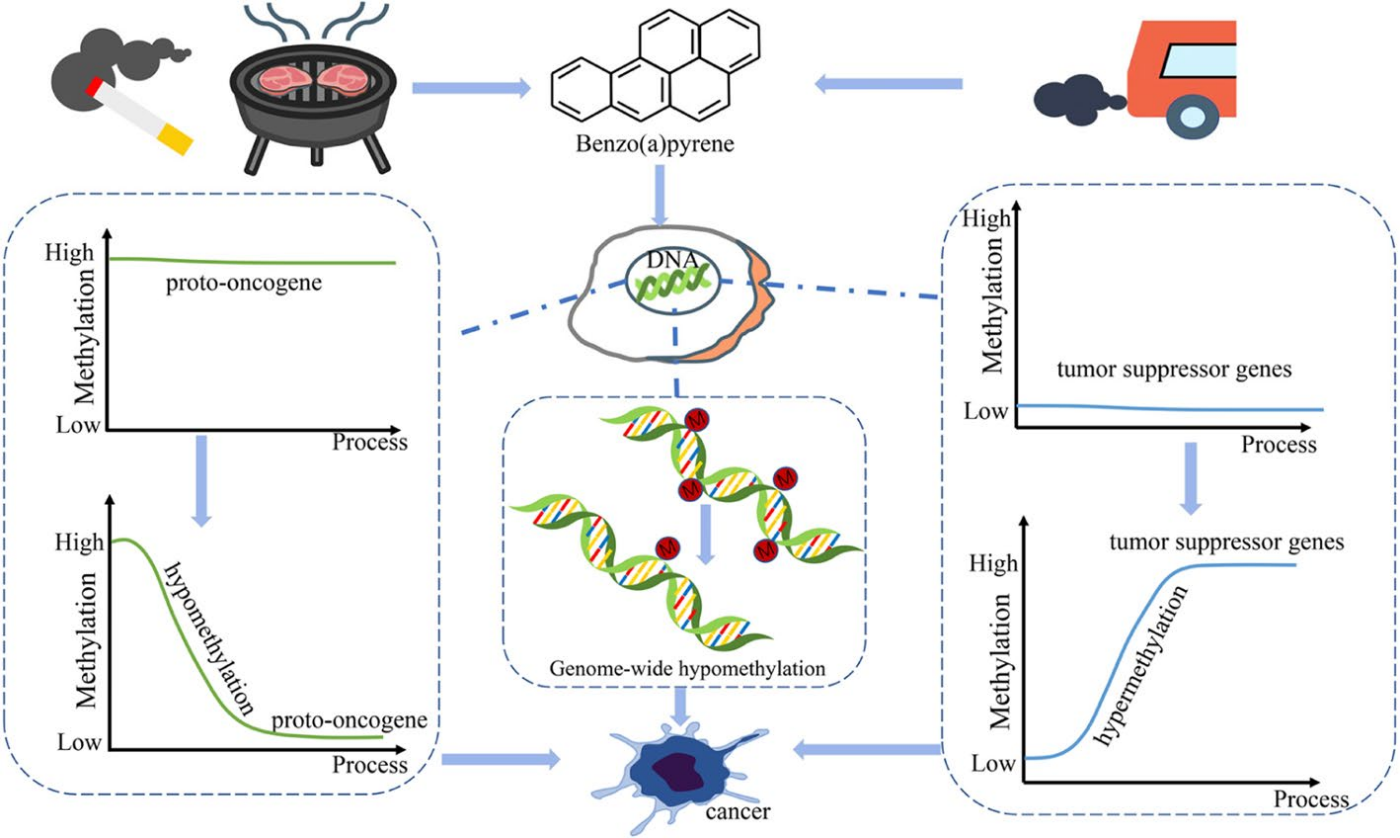

## Introduction

Benzo(a)pyrene (BaP) is the first recognized and most prototypical environ mental pollutants and carcinogens [6, 12, 63], Bukowska, Mokra et al. [14]. It comprises five fused aromatic rings, and derived from organic materials that has not been completely burned, including fossil fuels [73, 74] and timber [17]. Thus, BaP widely exists in automobile exhaust [111], deep-fried food [45], coal chemical combustion smoke and waste, etc.[68]. BaP can be stable in the environment due to its hydrophobicity [52] and chemical stability [101], while the collection of BaP in aquatic organisms is associated to its lipophilicity [2, 30], and it can accumulate along the food chain and enter the human body. Therefore, BaP can be detected in air (Schreiberová, Vlasáková et al. [123]), soil [108], water sources [53] and foods [159].

**Fig. 1:**
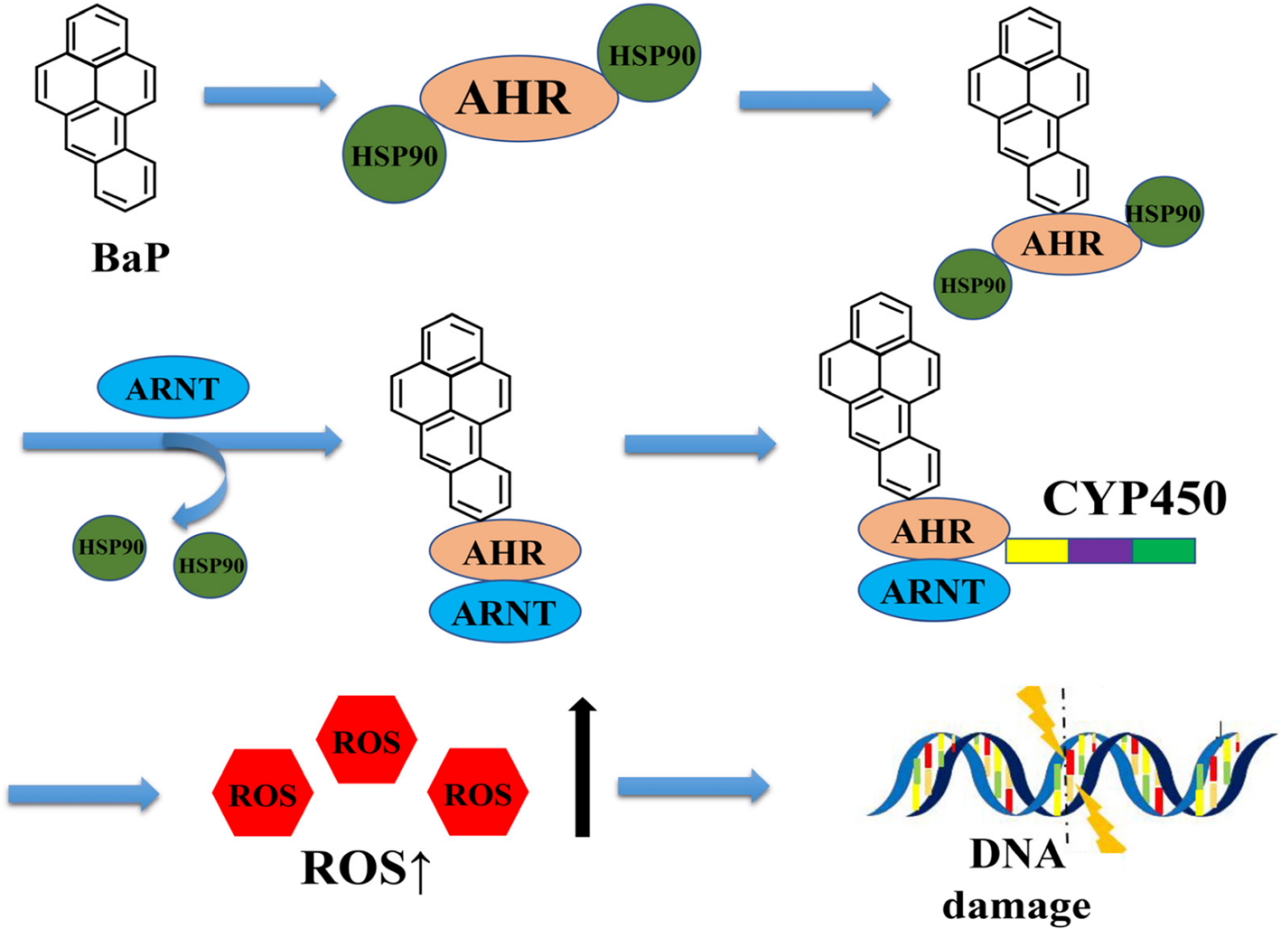
Oxidative stress of benzo(a)pyrene

As a byproduct of the development of science and technology, BaP is a great threat to human health. The International Agency for Research on Cancer (IARC) of the World Health Organization has listed BaP as a class I human carcinogen [92, 143], Goedtke, Sprenger et al. [46], and accumulated studies have revealed a close relationship between BaP and cancers in respiratory system [120], digestive system [44], reproductive system [121] etc. The toxicity of BaP to cells is mainly causing DNA damage [84] and oxidative stress [64, 65] by an increase in reactive oxygen species (ROS) production. BaP requires metabolic activation before reaction with DNA, and its metabolite benzo(a)pyrene-trans-7,8-dihydrodiol-9,10-epoxide (BPDE) induced genotoxicity by forming BPDE-DNA adduct [80, 106], which showing mutagenic and carcinogenic potential in cells [85]. Aryl hydrocarbon receptor (AHR) is a transcription factor [41], Gargaro, Manni et al. [43], when activated by BaP, AHR translocates to nucleus, and forms a heterodimer with the aromatic receptor nuclear transporter (Arnt) [61], which binds to the downstream target gene and activates the abnormal expression of cytochrome P450. P450 is one of the major ROS generators [96], Duan, Chen et al. [34], and ROS is a crucial factor of oxidative stress, which can induce oxidative DNA damage (Fig. 1) [91], cell apoptosis and even canceration [58, 102].

The initiation of cancer shares intimate links with genome epigenetic changes [29, 132], Hatano, Ideta et al. [54], in which DNA methylation and demethylation have been shown to be vital ways to cause carcinogenesis [47, 110, 155]. DNA methylation is the formation of 5-methylcytosine (5mC) by S-Adenosylmethionine (SAM) -dependent methyltransferases (DNMTs) (Martisova, Holcakova et al. [93], [149]. Baylin et al. suggested that the relationship between carcinogenesis and methylation was mainly through the following ways: firstly, the hypomethylation of the oncogene promoter. Secondly, the locally hypermethylation of the tumor suppressor gene promoter, and thirdly, 5mC-containing-DNA sequences or direct mutations exposed to ultraviolet light or other carcinogens [9]. DNA demethylation can either be passive or active [11], which is regulated by ten-eleven translocation (TET) family enzymes [113]. TET proteins belong to α-ketoglutarate- and Fe^2+^-dependent dioxygenases, and Tet1, Tet2 and Tet3 involved in this family [26]. Tet1 and Tet2 mainly regulates the level of 5-hydroxymethylcytosine (5hmC) in the promoter region of primordial germ cell (PGCs) genes or 5hmC inside PGCs gene, while Tet3-mediates paternal active DNA demethylation [7, 64, 65, 89, 94, 134, 161]. Active DNA demethylation occurs under the catalysis by TET proteins, which sequentially oxidize 5-methylcytosine (5mC) to 5hmC, then to 5-formylcytosine (5fC) and 5-carboxylcytosine (5caC) [38, 104, 126, 147]. In vivo experiments demonstrated that when the deletion of thymidine DNA glycosylase (TDG), which was pivotal in DNA demethylation, adult mice would develop delayed hepatocellular carcinoma (HCC) and hepatoblastoma (HB) (Onabote, Hassan et al. [103]). Thus, when DNA methylation and demethylation are abnormal, cells are likely to become cancerous.

It is general for cells to undergo epigenetic alterations after toxic treatment, but these changes include different trends and mechanisms. For example, NaAsO2 treatment of human bronchial epithelial (HBE) cells inhibits TET-mediated DNA demethylation and induces promoter hypermethylation of 8-oxoguanine DNA glycosylase (OGG1) and glutathione stransferase Pi 1(GSTP1) [139]. Hoang et al. found that pesticides can also cause DNA methylation changes [60]. Researchers have found that BaP changed genomic methylation levels in cells. In recent years, it has become a hot spot to reveal the carcinogenicity of BaP by changing genomic DNA methylation. Then how BaP as a carcinogen causes cancer through the regulation of epigenetics is worthy of consideration. Exploring the effects and mechanisms of BaP on genomic methylation will help understand BaP-induced carcinogenic mechanism, evaluate the risk of environmental pollutants, and provide an important theoretical basis for prevention of BaP from human health. In this review, we discuss the effects of BaP on DNA methylation and its correlation with carcinogenesis and provide future directions for researchers to reveal the mechanisms in these biological processes.

## DNA methylation in the presence of benzo(a)pyrene

### Benzo(a)pyrene and DNA methyltransferases

DNA methylation occurs when a methyl group is covalently attached to the 5th carbon position of CpG dinucleotides to form a product 5mC by DNA methyltransferase (DNMTs) (Martisova, Holcakova et al. [93]). DNMTs are mainly constituted of three structures: a C-terminal catalytic domain, an N-terminal regulatory domain and the central junction region [135]. Within the family of DNMTs, DNMT1, DNMT3a, and DNMT3b have DNA methyltransferase activities [20, 112]. DNMT1 sustains DNA methylation (Svedružić Ž [128]), DNMT3a and DNMT3b are responsible for de novo DNA methylation [76], Veiga, Lawrence et al. [136]). BaP induces DNA methylation through dysregulating the expression of DNA methyltransferases. It was reported that, the expression of DNMT3a was down-regulated when exposure of mouse embryonic fibroblasts to BaP for 2 weeks, while the expression of DNMT1 up-regulated after 4 weeks of exposure, and DNA methylation levels was elevated [152]. Moreover, BaP metabolite BPDE could induce DNMT3a binding to the promoters of related tumor suppressor genes, which resulted in the aberrant methylation of retinoic acid receptor-β2 (RAR-β2) in human esophageal cancer cells, and BPDE reduced DNMT3b expression [153].

However, in rainbow salmon liver, Bap was shown to inhibit the activity of DNA methyltransferase and decrease DNMT3a expression, which leading to DNA methylation globally reduce [77]. Moreover, compared with non-smokers, DNMT1 expression was apparently higher among smokers, and the level of methylated metabolites was also increased, which was associated with BaP in cigarette [71]. In human hepatic L02 cells, BaP at 0.1, 1 and 10 nmol induced the expression of DNMT1, DNMT3a and DNMT3b, resulting in glutathione-S-transferase-pi (GSTP) promoter region hypermethylation [131]. The above results demonstrated that the level of DNMTs could be modulated by BaP, which was one of the important ways to cause DNA abnormal methylation.

### Benzo(a)pyrene and gene methylation

BaP induces DNA methylation by targeting DNMTs, on the other hand, BaP can directly reduce the level of genome-wide methylation, and alter the methylation levels of specific genes, including tumor suppressor genes and proto-oncogenes, resulting in activation of proto-oncogenes or inactivation of tumor suppressor genes, and tumorigenesis [9].

#### Benzo(a)pyrene and genome-wide DNA methylation

As an epigenetic modifier, Bukowska et al. proposed that BaP reduces genomic DNA methylation by binding to DNA [15] the mechanism may be as follows: 1. BaP enters the human body through a series of reactions to form BPDE, which binds to DNA to inhibit the expression of DNMTs, thereby reducing the process of 5mC production and eventually causing a decrease in genome-wide methylation levels [160, 15]; 2. ROS generated by BaP can lead to oxidative DNA damage, which further affects the interaction between DNMTs and methyl CpG-binding proteins, thereby inhibiting the transcription of DNMTs, reducing the role of DNMTs in methylation, and ultimately reducing genome-wide methylation levels [57, 160], 3. BaP induces the conversion of SAM to SAH by increasing Glycine N-methyltransferase (GNMT) activity, reduces the covalent binding of SAM to a methyl group mediated by DNMTs to form 5mC, further reduces the production of 5mC, and causes a reduction in genome-wide methylation levels [37]. It was found that different concentrations of BaP (0.24, 2.4 and 24 μg/L) reduced the global levels of 5mC in Zebrafish embryonic cells, and BaP at 24 μg/L had the strongest activity [37]. In 16HBE, BaP was found significantly diminishing DNA methylation level throughout the genome in a time- and dose- dependent manner [62]. Similarly, when BaP was administrated at a dose of 600 mg/kg to the male ICR mice, the genome-wide DNA methylation level in blood and liver followed a downward trend along with the treatment time increasing [160]. Immunofluorescence staining results showed that the DNA methylation level of 16HBE cell treated with gradient concentration of BaP (2.5, 5, 10, 20 and 40 mmol/L) decreased by 3.43%, 9.27%, 23.76%, 32.55% and 43.15%, respectively [148], indicating that BaP inhibited genome-wide DNA methylation. Moreover, compared with normal tissue, genome-wide DNA in tumor tissue was found in a markly hypomethylated status (Ili, Buchegger et al. [66]). These results provide a theoretical foundation for illuminating the mechanism of carcinogenesis induced by BaP, which decreases the level of genome-wide DNA methylation, disturbs cell growth and apoptosis, and enables canceration.

#### Benzo(a)pyrene and specific gene methylation

Five to ten gene promoters hypermethylation when human immortalized bronchial epithelial cell treated with BPDE, including E-cadherin and Protocadherin-10 [28], which was related to breast cancer invasion [49]. Corrales et al. found that when Zebrafish embryos (during 96hpf) exposed to BaP, significant changes in methylation levels were observed in the promoters of 10 genes, including cancer-associated genes, metabolic genes, developmental and reproductive genes. Among them, six genes were hypermethylated including cancer-associated genes c-fos and MutL Homolog 1 (MLH1) [24], and four genes were hypomethylated. The results were consistent with the previous studies that cancer tissues expressed high level of hypermethylation in c-fos gene and MLH1 promoter [21, 86]. C57BL mice were treated with BaP at different concentrations (1.0, 2.5, 6.25 mg/kg), and DNA methylation in the promoter region of the N-methyl-d-aspartate receptor subunit 2B (NR2B) gene was up-regulated, and NR2B expression in prefrontal cortex and hippocampal part decreased by qPCR analysis, resulting in the diminishment of NR2B expression and abnormal behavior among mice [158]. By observing the different stages of 16HBE transformation induced by BaP, there were some correlations between the hypermethylation of the FMS-like tyrosine kinase-1 (FLT1) promoter and carcinogenesis of PAHs [56]. Liu et al. treated 16HBE with different concentrations of BaP (1, 2, 5 mmol/L) for 24 h, and related genes were measured by methylation-specific PCR. They found that GSTP1promoter methylation level was in negative correlation with BaP concentrations; BaP prohibited the methylation in the promoter of CYP1A1, which was the major members of the cytochromeP450 (CYP450) family [88]. BaP (5 µg/L) not only induced hypermethylation in the promoter region of tumor suppressor gene APC, but also induced demethylation in the proto-oncogene promoters of cyclooxygenase-2 (COX-2) and mutS homolog 2(MSH2) in normal peripheral blood mononuclear cells (PBMC) [156].

The above analyses show that BaP may induce a decrease in DNA methylation level accompanied by abnormal methylation patterns of some certain genes (Table 1), and the hypermethylation of tumor suppressor genes by Bap contributes to the occurrence and development of cancer.

**Table 1:** Methylation changes induced by benzo(a)pyrene

Changes in methylation		Sample type	Treatment dose / time	Correlation between dose / time and methylation	Reference
**Changes in DNMTs**	DNMT3a↓	Mouse embryonic fibroblasts	0.25 μmol 2 weeks, 4 weeks	Negative correlation	[152]
	DNMT1↑			positive correlation	
	DNMT1↑	Smokers	Not stated	Not stated	[71]
**Global methylation**	Whole genome↑	Mouse embryonic fibroblasts	0.25 μmol 2 weeks, 4 weeks	positive correlation	[152]
	Whole liver genome↓	Rainbow salmon liver	(1 ng/L), (10 ng/L) 24 h and 14 days	negatively correlated with time and dose	[77]
	Whole genome↓	Zebrafish embryos	0.24, 2.4, 24 μg/L	Negative correlation	[37]
		16HBE	10, 20, 40 μmol 24 h, 72 h	Negative correlation	[62]
		ICR mice	600 mg/kg	Negative correlation	[160]
**Hypermethylation of specific genes**	RAR-β2↑	Human esophageal cancer cells	24 h	Not stated	[153]
	GSTP↑	Human hepatic L02 cells	0.1, 1, 10 nmol	Positive correlation	[131]
	E-cadherin↑	Human Immortalized bronchial epithelial cells	0.05, 0.1, 0.25 μmol/L	Positive correlation	[28]
	Protocadherin-10↑			Positive correlation	
	c-fos↑	Zebrafish embryos	50 μg/L	Not stated	[24]
	MLH1↑			Not stated	
	NR2B↑	C57BL mice	1.0, 2.5, 6.25 mg/kg	Positive correlation	[158]
	FLT1↑	16HBE	20 mmol	Not stated	[56]
	APC↑	Human HCT116	5 μg/L	Not stated	[156]
**Hypomethylation of specific genes**	GSTP1↓	16HBE	1, 2, 5 mmol/L	Negative correlation	[88]
	CYP1A1↓			Negative correlation	
	COX-2↓	Human PBMC	5 μg/L	Not stated	[156]
	MSH2↓			Not stated	

### Carcinogenesis of benzo(a)pyrene by DNA methylation

**Fig. 2:**
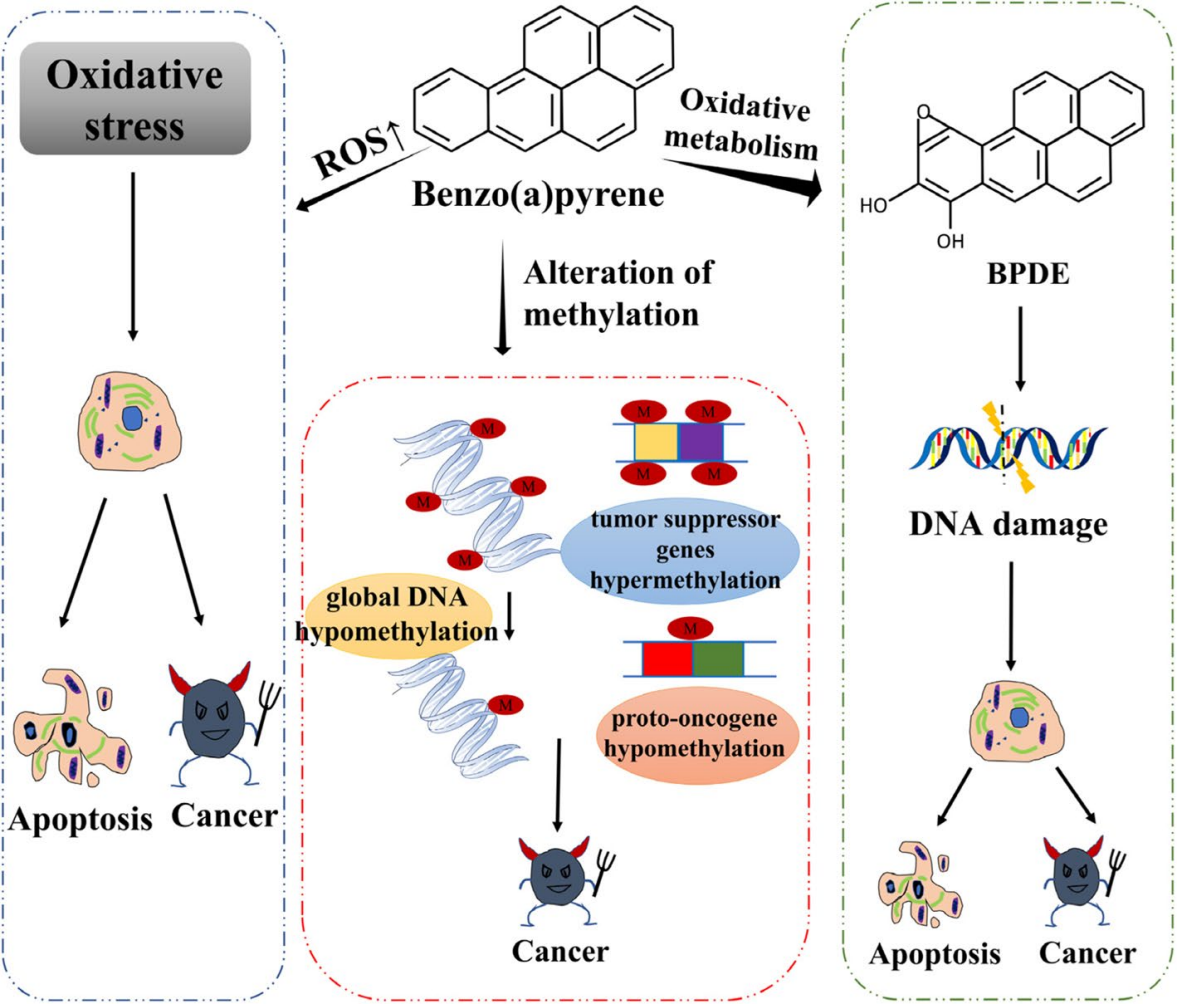
The mechanism of carcinogenesis of benzo(a)pyrene

Alteration of DNA methylation is the most representative epigenetic feature in cancer progression [47, 48, 72]. Silencing of tumor suppressor gene expression is caused by hypermethylation in gene promoter region, and genome-wide hypomethylation can lead to genome instability and proto-oncogene activation [146], eventually inducing the development of cancer [156]. The mechanisms of BaP can be summarized in two aspects: inducing hypomethylation of proto-oncogenes and activating them; and inhibiting tumor suppressor gene expression by hypermethylating them, both of which promote cancer initiation (Fig. 2).

## Association of benzo(a)pyrene methylation levels with multiple cancers

**Fig. 3:**
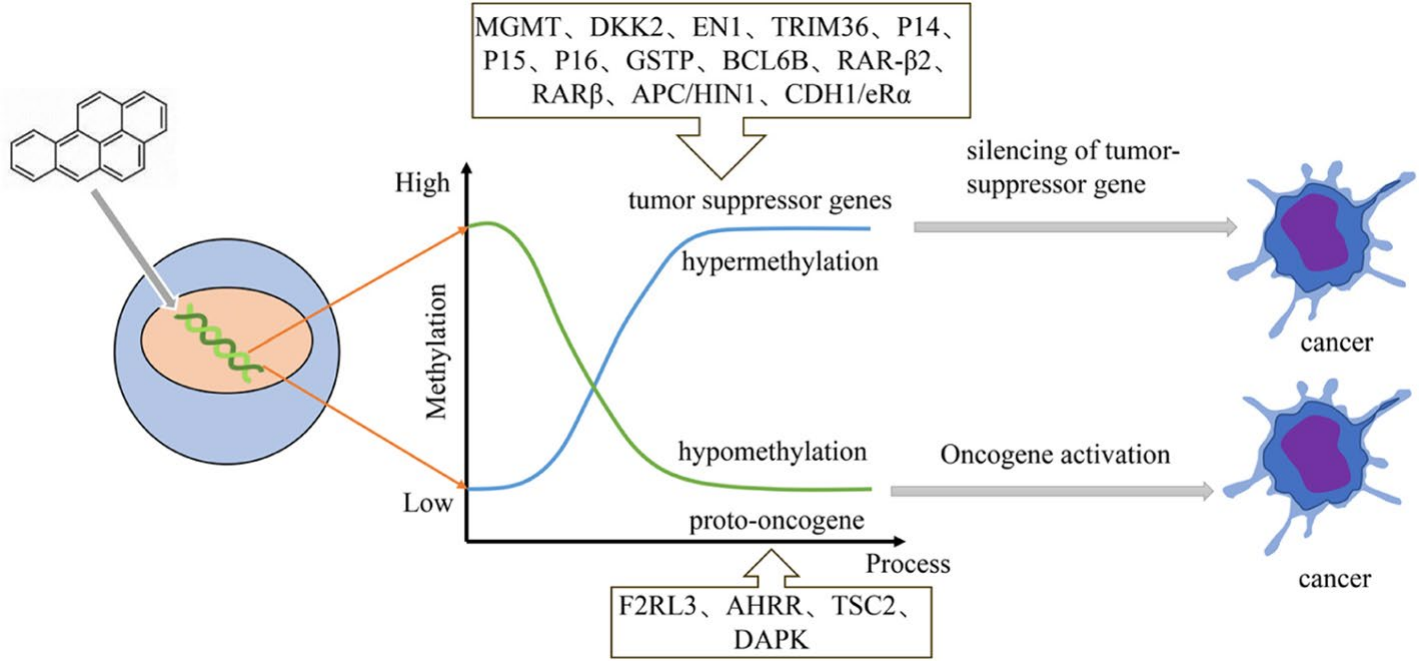
Benzo(a)pyrene alters gene methylation levels leading to cancer

BaP as a Class I carcinogen, can cause a variety of cancers [44, 120]. When BaP enters the human body, it undergoes a series of chemical reactions, and affects DNA methylation levels, which contributes to carcinogenesis of human respiratory system, digestive system and reproductive system, and eventually results in the occurrence of a variety of cancers (Fig. 3, Table 2).

**Table 2:** Carcinogenic epigenetic changes of benzo(a)pyrene

Changes in methylation		Objects exposed to BaP	Reference
**Genome-wide hypomethylation**		16HBE	[62, 148]
		zebrafish embryo	[37],
		ICR mouse	[160]
		colorectal cancer tumor tissue	[66]
**Hypermethylation of specific genes**	APC	human HCT116	[156]
	c-fos, MLH1	zebrafish embryo	[24]
	FLT1	16HBE	[56]
	DKK2, EN1	16HBE	[69]
	TRIM36	HBE	[55]
	P14, P15, P16	coke oven workers	[157]
	GSTP	lung Cancer Patients	[131]
	BCL6B	mice	[16]
	RAR-β2	human esophageal cancer cells	[127, 153]
	RARβ, APC	Breast cancer patients	[144]
	HIN1, CDH1	breast cancer patients	[145]
	eRα	mice	[117]
**Hypomethylation of specific genes**	GSTP1, CYP1A1	16HBE	[88]
	LINE-1, MGMT	workers exposed to PAHs	[33]
	F2RL3, AHRR	creosote-exposed workers, chimney sweeps	[3]
	TSC2	human MCF-7, human HCC1806	[115]
	DAPK-1	breast cancer cells	[145]

### Respiratory system cancer

Lung Cancer is one of the most common malignant tumors worldwide, with a highest mortality rate and morbidity rate [83, 133]. The occurrence of lung cancer is closely related to smoking [31, 67] and air pollution [122, 150], which are rich in BaP. Daily BaP concentrations were collected from 8 traffic stations in Barcelona during the severely cold period from 2013 to 2015. on the basis of Lung Cancer Risk (LCR) equation for BaP inhalation, the LCR values of the 8 stations exceeded the 10^−6^ threshold. It is concluded that chronic exposure to BaP increased the incidence of lung cancer [32]. Comparative study was conducted on lung cancer cases, and the results showed that the plasma BPDE-Alb adduct per SD (26.85 ng/mL) increased, the risk of lung cancer expanded by 46%. Meta-analysis determined that 15 CpG was interrelated with the plasma BPDE-Alb adducts, including Ubiquitin-conjugating enzyme E2 O (UBE2O), Sterile alpha motif domain containing protein 4A (SAMD4A), Acyl-CoA binding domain-containing 6 (ACBD6), Diacylglycerol kinase 2 (DGK2) and Schlafen 13 (SLFN13), which mediated the association between BaP exposure and 30%-60% lung cancer risk. These results highlighted the change of DNA methylation by BaP might be a contributing factor for lung cancer [95]. Some scholars believed that that BPDE, a metabolite of BaP, affected DNA methylation by binding to CpG, which was one of potential mechanisms of lung cancer induced by BaP [15]. The above studies indicated that BaP induced lung carcinogenesis by altering methylation of genes.

Long interspersed nucleotide element 1 (LINE-1) is the biggest family of long interspersed nucleotide elements, and some studies have shown that LINE-1 is an indicator of methylation levels in the genome [119]. Numerous tumors have been reported to exhibit the hypomethylation of LINE-1 [105]. The methylation levels of LINE-1 and O6-methylguanine-DNA methyltransferase (MGMT) in PAHs exposure group and control group were detected by pyrosequencing (PSQ) technology, and the results demonstrated that PAHs induced LINE-1 hypomethylation, and genome-wide hypomethylation might promote genomic instability, eventually contributed tumor progression; MGMT promoter hypomethylation resulted in the abnormal expression in gene level, which reduced its ability to repair damaged genes, further exacerbating the stability of the chromosomes. According to these findings, PAH-induced carcinogenesis seemed to be mediated by specific methylation in the CpG island region of the MGMT [33]. These results were consistent with the situation of LINE-1 hypomethylation [107] and MGMT promoter hypomethylation [59] in lung cancer cells. A comparative study was utilized to detect DNA methylation in 16HBE cells treated with BaP and in lung cancer Xuanwei lung cancer (XWLC) cells without any treatment. The results showed that low levels of 5mC and high levels of 5-hmC were found in XWLC cells, and lower global 5-mC level and higher 5-hmC level were found in BaP-treated 16HBE cells, which suggested that BaP treatment led to cell demethylation and BaP-induced alterations in DNA methylation might be a contributing factor to aberrant DNA methylation in XWLC. Moreover, Bisulfite sequencing PCR (BSP) analyzed the state of methylation of 25 CpG dinucleotides located in the promoter region of Dickkopf-2 (DKK2) and 20 CpG dinucleotides in the Engrailed 1 (EN1) promoter region after 16HBE cell treated with BaP, and the results showed that hypermethylation occurred at the, 1^st^, 2^ed^, 5^th^ and 6^th^ CpG dinucleotides in DKK2 promoter region and the 1^st^, 8^th^ and 14^th^ CpG dinucleotides in the EN1 promoter element, which were similar to those observed in XWLC cells [69]. Based on these findings, a mechanism on how BaP-induced DNA methylation changes resulting in lung cancer was explored. BaP induced significant hypermethylation of the DKK2 and EN1 gene promoter elements, and then inhibited DKK2 and EN1 gene expression, which promoted lung cancer cell proliferation and cancer development.

In 2017, He et al. observed in HBE cells that following exposure to BaP, a tumor suppressor gene, tripartite motif containing 36 (TRIM36) were hypermethylated, which may be involved in BaP-induced cell carcinogenesis. To prove this conjecture, pyrosequencing technologies were applied to detect the degree of TRIM36 methylation in non-small cell lung cancer (NSCLC) patients. The results showed that TRIM36 hypermethylation was found in 90.0% (27/30) of the NSCLC, indicating that BaP induced hypermethylation of TRIM36, then promoted lung carcinogenesis [55]. In 2019, the same study group found that hypermethylation of FLT1 promoter, a tumor suppressor gene, probably involved in the carcinogenic process of PAHs [56]. Both studies demonstrated that BaP induced part of tumor suppressor genes inactivation through hypermethylation, which was associated with the development and progression of cancer.

Studies have proven that hypermethylation in the promoter region of P14 (ARK), P16 (INK4a) gene contributed to the occurrence of lung cancer, which were considered to be an early event in lung carcinogenesis [130, 151]. Coke oven workers are at high risk of developing lung cancer, because of the high concentration of BaP in the working environment [25, 109]. By comparing the concentration of BaP in the air of coke oven workshop with control workshop, Zhang et al. found that the average concentration of BaP at the coke oven top (1286.5 ng/m^3^) was 147 times higher than the average concentration (8.6 ng/m^3^) in control workshop, which suggested that BaP played a major role in lung cancer development in coke-oven workers. To explore the mechanism of carcinogenesis in BaP, the author chose 74 coke-oven workers long-time exposing to BaP as experimental group, and 47 plumbers who had less opportunity exposing to BaP as control group, and genomic DNA methylation from workers' PBMC were measured. The results showed that CpG island in the promoter regions of tumor suppressor genes P14 (Ark), P15 (INK4b) and P16 (INK4a) were significantly higher in experimental group [157]. It is well known that silencing of tumor-suppressor genes is associated with the promoter-region hypermethylation, which can induce cell proliferation and over-growth, and ultimately lead to tumorigenesis. Thus, it may be the main reason for coke-oven workers at high prevalence rate of lung cancer.

DNA hypomethylation in factor II receptor-like 3 (F2RL3) and aryl hydrocarbon receptor repressor (AHRR) gene is smoking-related biomarker in blood, which closely correlated with lung cancer incidence and mortality. Alhamdow et al. [3] compared the DNA methylation of lung cancer-related genes F2RL3 and AHRR between the workers occupationally exposed to PAHs and the control group. The CpG methylation levels of AHRR and F2RL3 in the exposed group were significantly lower than those in the control group, which was consistent with the above expression.

### Digestive system cancer

Gastric cancer (GC) is one of the most commonly malignant cancers [19, 82]. The common causes of GC are H. pylori infection [23], chronic atrophic gastritis [78], unhealthy diets [129] and inheritance [22] et al. China has a high incidence rate of liver cancer [36], and viral infection [79], chronic alcoholism [50], eating moldy and deteriorate foods [116] can lead to hepatic cancer. Moreover, BaP can also induce gastric and hepatic cancer [44, 140]. It has been shown that the promoter region in GSTP gene (a tumor suppressor gene) was significantly hypermethylated in HCC patients, and BPDE-Alb adducts were remarkably correlated with GSTP methylation level. In addition, there was a higher risk of developing HCC in those individuals with higher levels of BPDE-Alb adducts and GSTP hypermethylation. Tian et al. further evaluated the association between epigenetic alterations caused by BaP and the risk factors in HCC. The results showed that BaP induced the hypermethylation of promoter regions in the detoxification gene GSTP, leading to a loss of protective function owing to gene silencing, which triggered the accumulation of poisons in liver, increased oxidative stress, DNA damage and hepatocarcinogenesis [131]. B-cell CLL/lymphoma 6 member B (BCL6B) is a tumor suppressor. Once the promoter regions of BCL6B is excessively methylated, gene expression is suppressed or silenced, leading to colonic carcinoma [51], and gastric carcinogenesis [16, 87]. To explore how BCL6B functions as a tumor suppressor gene during gastric carcinogenesis, Cai et al. used Bcl6b-deficient mice and wild type mice to investigate Bcl6b's role in the development of gastritis and GC caused by BaP. The results showed in wild mice that during gastric carcinogenesis induced by BaP, Bcl6b expression was gradually decreased by its promoter CpG islands hypermethylation, alongside an increased in inflammatory response. In addition, in BCL6B gene knockout mice, BaP induced inflammatory response and promoted gastric carcinogenesis [16]. Thus, BaP regulated the expression and function of BCL6B through promoter hypermethylation, then triggered an elevated inflammatory response which promoted the occurrence and development of tumors.

At present, absent expression of Retinoic Acid Receptor-β2 (RAR-β2) and hypermethylation in its promoter region have been used as diagnostic markers of oncogenesis [98]. RAR-β2 promoter hypermethylation is an early event during esophageal cancer progression [142]. It was found that RAR-β2 promoter hypermethylation was induced by BPDE, and then its expression was decreased by recruiting DNMT3A in combination with RAR-β2, which promoted the occurrence and development of esophageal cancer [153]. The inhibition of RAR-β2 expression by BPDE induced COX-2 highly expression [127], and overexpression of COX-2 was linked to esophageal cancer [4, 18]. There are some similarities between the mechanisms on lung cancer and digestive system cancer induced by BaP. BaP modulates aberrant DNA methylation or promoter hypermethylation in various tumor suppressor genes, which alters gene expression and contributes carcinogenesis.

### Reproductive system cancer

breast cancer is a common malignancy in women (Sethi, Shanmugam et al. [125], Velloso, Trombetta-Lima et al. [137]). Its etiology is related to genetic factors [97], endocrine hormones [1], and exposure to environmental pollutants such as BaP [5]. There is evidence that aberrant methylation of genomic DNA contributes to breast cancer development [39], Lubecka, Kaufman-Szymczyk et al. [90], [35]. Therefore, BaP inducing breast cancer through abnormal DNA methylation is worth investigating.

In 2004, Sadikovic et al. treated MCF-7 and MDA-MB 231 cell lines by 5 mol/L of BaP, and found that the level of global genome methylation was reduced by 12% in BaP-treated cells [114]. Previous studies have shown that genome-wide hypomethylation was observed in breast cancer [100], which was consistent with Sadikovic’s study. In 2006, the authors tested the growth dynamics of four breast cancer cell lines exposed to BaP, and found that BaP exposure reduced cell proliferation via accumulation of cell cycle at S and G2/M phases, and induced p53-dependent cellular apoptosis. Amplification of inter-methylated sites (AIMS) analysis showed that BaP induced the hypomethylation of tumor suppressor gene subunit 2 (TSC2) in human MCF-7 and HCC1806 cells [115], which also could be detected in breast cancer [70]. All the results indicated that p53-specific cell cycle interruption and DNA methylation disruption were resulted from BaP exposure, and short interspersed nucleotide elements (SINEs) acted as specific targets on the association of Bap exposure with DNA methylation, which contributed to genomic instability and breast carcinogenesis.

It is suggested that exposure to environmental contaminants correlated with DNA methylation and breast cancer progression. A study on the relationship among DNA methylation, PAH-DNA adducts, and breast cancer was carried out in 2015 by White et al. Thirteen genes were identified associated with breast cancer occurrence, and that the tumor suppressor gene retinoic acid receptor beta (RARβ) and the adenomatous polyposis coli (APC) promoter specific methylation interacted with PAH-DNA adducts, which affected the hormone receptor expression, and increased the risk for developing breast cancer [144]. It is more likely to express the hypermethylation of RARβ and APC in the promoter regions in breast cancer tissue in comparison to normal breast tissue [81].

The changes of promoter methylation in death-associated protein kinase (DAPK) [27], harpin-induced 1(HIN1) [99]and Cadherin 1 (CDH1) [138] genes can be acted as biomarkers for carcinogenesis. In 2016, White et al. discussed methylation in the promoter region of cancer-related genes and global methylation in the peripheral blood of breast cancer patients with long-time exposing to PAHs. They found that the expression level of the tumor suppressors HIN1 and CDH1 were elevated, and the methylation level of death-associated protein kinase 1 (DAPK-1), a breast tumorigenic gene, was decreased. Moreover, an increased frequency of chromosomal mutations and instability were observed in peripheral blood with LINE-1 hypomethylation [145]. The results indicated that air pollutants such PAHs induced mammary tumor formation by altering the methylation of oncogenes [118]. In 2021, Sahay proved that prenatal exposure to PAHs induced the hypermethylation of CpG-2012 and CpG-2138 in estrogen receptors α (ERα) gene promoter region, reducing the expression of eRα at gene and protein levels in mouse mammary gland. Moreover, PAHs could inhibit the expression of tumor suppressor gene Brca, and eventually induced mammary carcinogenesis [117]. As the most representative carcinogen in PAHs [8, 42, 124], BaP contributed to breast cancer occurrence by changing tumor suppressor genes methylation.

## Discussion

Overall, BaP as the product of combustion of organic matter including fossil fuels, can pollute the environment. Generally, BaP can be found in soil [154], air [75] and water sources [141].Because of its chemical properties [2, 52, 101], BaP can enter human body and cause the incidence of disease in human. As a common toxicant, BaP triggers cell damage and cancerization by forming DNA-adduct, oxidative stress, or in the epigenetic aspect. In this review, the changes of cellular genomic methylation levels modulated by BaP were summarized. It is commonly believed that BaP reduced genome-wide methylation levels. However, Yauk et al. [152] study found that BaP induced genome-wide hypermethylation by cytosine extension assay in 2008, which might be related to the insufficient techniques. Cytosine extension assay is mainly used for testing the overall level of methylation of CpG, CpHpG, CNpG and asymmetric sites in plant tissues [10, 13], but the research objects of Yauk are animal cells, which may contribute to the sharp contrast results.

BaP as a PAHs carcinogen, enters human body through diet, cigarette smoke and gasoline exhaust, and promotes carcinogenesis in human organs and tissues. In this review, mechanisms of BaP-induced carcinogenicity were discussed at the epigenetic level. GSTP is a member of the tumor suppressor gene family, the studies of Tian et al. and Liu et al. gained different results. Tian et al. found that BaP induced hypermethylation of GSTP, but Liu et al. showed that BaP caused hypomethylation of GSTP1. The differences on the dosage of BaP and exposure time might contribute to these different results. The epigenetic toxicity of BaP was not limited to methylation and demethylation, studies have shown that BaP promoted histone acetylation and deacetylation [15, 40], which could in turn lead to abnormal chromatin structure and aberrant gene expression. The different results from Tian and Liu could be caused via other epigenetic mechanisms. It is still not well-known that BaP inducing abnormal DNA methylation associated with digestive system cancer and reproductive system cancer, but there is certain correlation between BaP exposure and tumor suppressor gene methylation in these two cancers. The epigenetic changes of genes induced by BaP and the mechanisms of carcinogenesis have not been fully elucidated, and needed a deeper understanding.

BaP is a carcinogen and can cause epigenetic changes such as DNA methylation and histone acetylation [15], in addition, BaP can affect DNA methylation and demethylation, the most important part of epigenetics, by influencing DNMTs and thus DNA methylation [77, 152]. Based on the above, it is known that DNMTs with DNA methyltransferase activity mainly include DNMT1, DNMT3a and DNMT3b, and BaP further affects genome-wide or specific gene methylation levels by increasing DNMT1 expression or decreasing DNMT3a expression [71, 152]. However, the mechanism of how BaP affects gene methylation changes by altering DNMTs is still unclear, and the enzymes involved in the methylation and demethylation process include, in addition to DNMTs, the TET protein family, which can mediate the DNA demethylation process [147]. Whether BaP causes DNA methylation changes with the involvement of TET proteins is less reported in the relevant literature. Therefore, it is important to further explore the mechanism of BaP-induced genome-wide methylation level reduction, proto-oncogene hypermethylation and oncogene hypomethylation, to discover the cause of cancer at the molecular level, to solve this puzzle by scientific means, and to provide treatment options for patients who develop cancer due to BaP exposure, and to make a significant contribution to human health. Therefore, it is urgent to explore the mechanism of how BaP affects gene methylation changes by affecting DNMTs.

Nowadays, with the development and progress of industrialization, air pollution caused by atmosphere BaP has become a problem and demands prompt solution. Cancer caused by BaP has also become one of the threatening factors for human health. Governments should not only reduce the emission of BaP to the atmosphere, but also demand further study the mechanisms on the carcinogenesis by epigenetic modification. Folic acid plays an extremely important role in human health. In addition to effectively preventing diseases such as neonatal neural tube defects and megaloblastic anemia, folic acid can provide methyl donors to participate in the transfer of one carbon unit. DNA methylation is a process in which DNMT catalyzes the covalent binding of SAM with a methyl to form 5mC. According to the above, we know that BaP can reduce the level of methylation of the whole genome, and then cause cancer, so whether folic acid, as a methyl donor, can further promote the occurrence of DNA methylation, effectively alleviate the decrease of the level of methylation of the whole genome, and then reduce the incidence of cancer is worthy of further exploration in the future. Whether 5-azacytidine (5-Aza), as an inhibitor of DNA methyltransferase, can inhibit cancer caused by the activation of BaP-induced hypomethylation of proto-oncogenes has not been reported, which is also the direction that scientists will study in the future. Finding suitable drugs to act on specific targets to reduce the incidence of cancer caused by exposure to environmental poisons, is the significance of scientific existence, but also the direction of researchers' efforts. In order to provide new insights on reducing or even eradicating the harm of BaP to public health, better maintenance of general public health should be devoted to the development of prevention.

## Data Availability

Not applicable because of no datasets and materials were generated or used during the current study.

## Abbreviations

BaP: Benzo(a)pyrene
PAHs: Polycyclic aromatic hydrocarbons
IARC: The International Agency for Research on Cancer
ROS: Reactive oxygen species
BPDE: Benzo(a)pyrene-trans-7,8-dihydrodiol-9,10-epoxide
AHR: Aryl hydrocarbon receptor
Arnt: Aromatic receptor nuclear transporter
TET: Ten-eleven translocation
5mC: 5-Methylcytosine
5hmC: 5-Hydroxymethylcytosine
5fC: 5-Formylcytosine
5caC: 5-Carboxylcytosine
PGCs: Primordial germ cell
TDG: Thymidine DNA glycosylase
HCC: Hepatocellular carcinoma
HB: Hepatoblastoma
DNMTs: DNA methyltransferase
SAM: S-Adenosylmethionine
16HBE: Human bronchial epithelial cells
OGG1: 8-Oxoguanine DNA glycosylase
RAR-β2: Retinoic acid receptor-β2
MLH1: MutL Homolog 1
NR2B: N-methyl-d-aspartate receptor subunit 2B
FLT1: FMS-like tyrosine kinase-1
CYP450: CytochromeP450
GSTP: Glutathione-S-transferase-pi
GNMT: Glycine N-methyltransferase
GSTP1: Glutathione S‑transferase P1
COX-2: Cyclooxygenase-2
MSH2: MutS homolog 2
PBMC: Peripheral blood mononuclear cells
LCR: Lung Cancer Risk
UBE2O: Ubiquitin-conjugating enzyme E2 O
SAMD4A: Sterile alpha motif domain containing protein 4A
ACBD6: Acyl-CoA binding domain-containing 6
DGK2: Diacylglycerol kinase 2
SLFN13: Schlafen 13
LINE-1: Long interspersed nucleotide element 1
MGMT: O6-methylguanine-DNA methyltransferase
PSQ: Pyrosequencing
XWLC: Xuanwei lung cancer
BSP: Bisulfite sequencing PCR
DKK2: Dickkopf-2
EN1: Engrailed 1
TRIM36: Tripartite motif containing 36
NSCLC: Non-small cell lung cancer
F2RL3: Factor II receptor-like
AHRR: Aryl hydrocarbon receptor repressor
GC: Gastric cancer
BCL6B: B-cell CLL/lymphoma 6 member B
AIMS: Amplification of inter-methylated sites
SINEs: Short interspersed nucleotide elements
TSC2: Subunit 2
RARβ: Retinoic acid receptor β
APC: Adenomatous polyposis coli
DAPK: Death-associated protein kinase
HIN1: Harpin-induced
CDH1: Cadherin 1
DAPK-1: Death-associated protein kinase 1
Erα: Estrogen receptors α
5-Aza: 5-Azacytidine
